# DNA Helicases as Safekeepers of Genome Stability in Plants

**DOI:** 10.3390/genes10121028

**Published:** 2019-12-10

**Authors:** Annika Dorn, Holger Puchta

**Affiliations:** Botanical Institute, Molecular Biology and Biochemistry, Karlsruhe Institute of Technology, Fritz-Haber-Weg 4, 76131 Karlsruhe, Germany; annika.dorn@kit.edu

**Keywords:** helicase, DNA repair, DNA recombination, meiosis, crossover, gene targeting

## Abstract

Genetic information of all organisms is coded in double-stranded DNA. DNA helicases are essential for unwinding this double strand when it comes to replication, repair or transcription of genetic information. In this review, we will focus on what is known about a variety of DNA helicases that are required to ensure genome stability in plants. Due to their sessile lifestyle, plants are especially exposed to harmful environmental factors. Moreover, many crop plants have large and highly repetitive genomes, making them absolutely dependent on the correct interplay of DNA helicases for safeguarding their stability. Although basic features of a number of these enzymes are conserved between plants and other eukaryotes, a more detailed analysis shows surprising peculiarities, partly also between different plant species. This is additionally of high relevance for plant breeding as a number of these helicases are also involved in crossover control during meiosis and influence the outcome of different approaches of CRISPR/Cas based plant genome engineering. Thus, gaining knowledge about plant helicases, their interplay, as well as the manipulation of their pathways, possesses the potential for improving agriculture. In the long run, this might even help us cope with the increasing obstacles of climate change threatening food security in completely new ways.

## 1. Introduction

**Figure 1:**
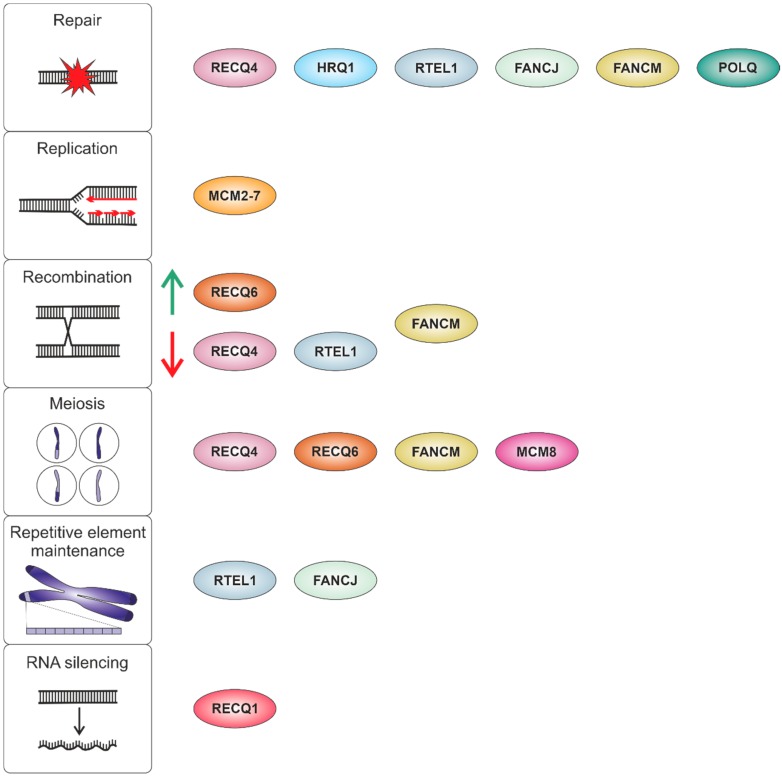
Overview of DNA helicases involved in safeguarding plant genome stability. The figure depicts in vivo functions identified so far for DNA helicases that are described in this review.

DNA helicases are motor proteins that catalyze the unwinding of double-stranded DNA by using the energy of ATP hydrolysis. As DNA helicases are indispensable for the most basic processes of genetic information processing, including replication, transcription, repair and recombination, these enzymes are highly conserved not only between species but also between kingdoms. Of special interest is the role of DNA helicases involved in safeguarding genome stability. Genomes not only differ drastically in size but also in composition. Moreover, a long living multicellular eukaryote faces other genomic threats than a quickly dividing unicellular prokaryote. Thus, the tasks helicases face in these organisms differ considerably. Despite all these differences, there is a certain functional and genetic conservation when it comes to safeguarding genomes. Orthologs of the same kind of helicase are often found in different kingdoms. However, they might vary in their individual number and their detailed functions. This became clear by comparing studies performed in the last 30 years in yeast and in mammals. Here, we want to sum up recent research performed in plants on this enzyme class (Figure 1). It turns out that in plants, besides a basic conservation of functions with yeast as well as mammals, interesting peculiarities can also be found. 

## 2. RecQ Helicases

RecQ helicases are central for the maintenance of genome stability in all domains of life. This reflects in the presence of at least one homolog in each organism, while the number of RecQ helicases rises with the complexity of the genome. While baker’s yeast holds only one classical RecQ homolog called Sgs1, five different RecQ helicases could be identified in humans. Plants also possess multiple RecQ homologs forming a helicase family, which contains up to seven members [1]. Recent phylogenetic analyses could uncover the relationship of plant RecQ helicases and their human orthologs [2] (Table 1). The initial identification of RecQ helicases in plants lead to the consecutive numbering of the individual proteins, however this nomenclature differs between humans and plants. In the following, we will refer to the plant nomenclature in respect of RecQ subfamilies. The RecQ family harbors six subfamilies, whereby the five human RecQ helicases are each associated with individual subfamilies (RecQ2–6). The conserved presence of homologs from the RecQ2, RecQ4, and RecQ5 subfamilies in all plant clades was furthermore shown. *Arabidopsis thaliana* possesses orthologs of all subfamilies, with a *Brassicaceae* specific duplicated RecQ4 homolog. No direct RecQ6 homolog is present in *Arabidopsis* but RECQsim was suggested to originate in the RecQ6 subfamily. The moss *Physcomitrella patens* does not encode RecQ1 or RecQ3 homologs, but a RecQ6 and a duplicated RecQsim representative are present. In the following sections we want to give an overview about the functions of the different RecQ helicases in plants.

### 2.1. RECQ4 Homologs and the RTR Complex

Probably the best-characterized helicase in plants is the RecQ helicase RecQ4 and its homologs. The human representative of the RecQ4 subfamily BLM is a major factor in the maintenance of genome stability. Mutations in the *BLM* gene are associated with the hereditary disease Bloom’s syndrome, characterized by an increased susceptibility to cancer and growth retardation [3]. On the cellular level, a distinct genome instability paired with an elevated homologous recombination (HR) rate, resulting in a high number of sister chromatid exchanges was reported [4]. As member of the *Brassicaceae* family, *Arabidopsis* harbors two RecQ4 homologs, RECQ4A and RECQ4B which have undergone a subfunctionalisation so only RECQ4A acts as a true functional BLM homolog [5,6]. This was already underlined with the first studies regarding RECQ4A, where ectopic expression of At*RECQ4A* in yeast *sgs1* mutants was able to complement genotoxin hypersensitivity and hyperrecombination [7]. At*recq4A* mutants also depict hypersensitivity towards a wide variety of genotoxins, including crosslinking and methylating agents. Furthermore, the characteristic hyperrecombination phenotype is conserved [6,7]. This directly implies the suggested role for RecQ4 homologs in DNA repair and in processing recombination intermediates towards non-crossover (NCO) outcomes. In a biochemical approach, the ATP-dependent helicase activity of AtRECQ4A could be confirmed. Furthermore, AtRECQ4A was shown to promote replication fork regression, indicating a role for the helicase in resolving stalled replication forks [8]. The function of RECQ4A in response to replicative stress is dependent on both its helicase function and the N-terminus, while both domains likely mediate two independent subfunctions in crossover (CO) suppression [8]. The important role of AtRECQ4A in processing complex DNA structures is further underlined with the synthetically lethal phenotype of *recq4A mus81* double mutants [9]. Here, most likely the simultaneous absence of RECQ4A and the repair endonuclease MUS81 lead to the accumulation of unresolved replication intermediates that arise during recombination, as the additional suppression of HR was able to rescue this drastic phenotype [10]. Thereby a major role of RECQ4A in the SDSA pathway was specified. AtRECQ4A is additionally an important player in the repair of DNA-crosslinks (CLs), where it defines one of the three main pathways in intrastrand CL repair [10]. A hidden function of AtRECQ4A in interstrand CL (ICL) repair could be revealed with the analysis of double mutants with the helicase AtFANCM [11]. Further research could classify AtRECQ4A into repair pathways for both inter- and intra-strand CLs [12,13,14]. Detailed information about CL repair in plants, the interplay of the different factors and the role of RECQ4A was recently reviewed in [15]. Analyses in rice and *P. patens* could verify the functions of plant RecQ4 homologs in DNA repair and HR suppression [2,16,17]. AtRECQ4B, on the other hand, was not connected to somatic DNA repair so far and a CO promoting role has been proposed [6]. The functional difference between the two RecQ4 homologs was suggested to depend on a differentiation in the N-terminus of both helicases as a chimeric RECQ4A variant containing the RECQ4B C-terminus was able to complement all relevant *recq4A* mutant phenotypes, while this was not possible with a swap in the N-terminus [8]. Cross-species complementation analyses with RecQ4 homologs from *Arabidopsis* and *P. patens* support the functional conservation between AtRECQ4A and RecQ4 homologs from species where only one homolog is present. Thereby the expression of At*RECQ4A* in Pp*recq4* mutants could rescue the mutant phenotypes and vice versa, while this was not the case for At*RECQ4B* [2]. The important role of AtRECQ4A in somatic DNA repair and HR raised the question of a role for the helicase in meiotic double-strand break (DSB) repair. Indeed, loss of AtRECQ4A leads to a fertility defect with connections of non-homologous chromosomes via telomeric bridges [18]. This was the first hint to an important meiotic function of AtRECQ4A. As *P. patens recq4* mutants exhibit a striking fertility defect, furthermore associated with developmental aberrations, this meiotic role appears to be evolutionary conserved [2]. Astonishingly, recent research highlighted a key role for both *Arabidopsis* RecQ4 homologs in meiotic CO regulation. Here, the concurrent mutation of *RECQ4A* and *RECQ4B* was shown to result in a 6-fold increase in meiotic COs, while the number of DSBs and chromosome segregation were unaffected [19]. Thereby, a function for both helicases in the suppression of noninterfering COs was demonstrated. Lately, this could be confirmed in crop plants as a biallelic *recq4* knockout led to a 1.8-fold CO increase in an interspecific tomato hybrid between *Solanum lycopersicum* and *S. pimpinellifolium* [20]. Furthermore, similar results could be obtained for pea and rice hybrids [21]. This is in line with experiments regarding gene targeting (GT) in *P. patens*, where a strong inhibiting effect of RECQ4 was determined [2]. The utilization of this CO frequency and GT enhancement in plant breeding is further discussed in Section 5.

The interaction partners of RECQ4A are also of special interest, as it is acting in the evolutionary conserved RTR complex consisting of the RecQ helicase, the Topoisomerase 3α and the structural proteins RMI1 and RMI2 [22]. The presence of all complex partners in *Arabidopsis* was confirmed and the proteins were shown to interact in vivo [6,19,23,24,25]. The central function postulated for the complex is the dissolution of recombination intermediates like double Holliday junctions (HJs) to a NCO outcome. In this dissolution, the RecQ helicase pushes the two junction points together via its helicase activity to form a hemicatenane intermediate. This structure can then be cleaved by the topoisomerase of the complex. The structural RMI proteins are essential for this reaction and stimulate complex formation [26,27,28]. The role of the complex seems to be conserved in plants, as *Arabidopsis* mutants for all partners exhibit corresponding phenotypes with hyperrecombination and genotoxin hypersensitivity. Furthermore, interesting additional features for the complex were identified. AtTOP3α was shown to be essential for the dissolution of replication-associated recombination intermediates in the absence of the endonuclease AtMUS81, similar to the function of AtRECQ4A. Thereby, it was postulated that the C-terminal zinc finger domains of TOP3α target the protein to HJ-like recombination intermediates [29]. Astonishingly, AtTOP3α, and AtRMI1 possess essential functions in meiotic recombination as meiotic catastrophe occurs in mutant lines of both genes [23,24]. Thereby, most likely unresolved recombination intermediates accumulate, leading to chromatin bridges and fragmentation. This indispensable meiotic role of RMI1 was shown to rely on the N-terminus of the protein while both RMI1 and TOP3α additionally seem to form a subcomplex that is specifically involved in meiotic CO suppression, via their C-terminal domains [30,31]. AtRMI2 was shown to be involved in the maintenance of 45S rDNA copy number, whereby the simultaneous loss of RMI2 and the helicase RTEL1 resulted in a copy number diminished to about a third [25]. This was an exciting new finding as a role in rDNA stability has not been reported for other eukaryotes, further underlining the indispensable function of the RTR complex for maintaining genome stability in multiple ways.

### 2.2. Other Classical RecQ Helicases

The specific functions of the other RecQ family members in plants remain mostly obscure. The biochemical features of the RecQ2 and RecQ3 homologs from *Arabidopsis thaliana* were characterized, while no in vivo function could be demonstrated so far. For both proteins an ATP-dependent 3’–5’ helicase function was demonstrated, however they seem to act on different substrates. While AtRECQ2 processes various D-loop structures and displays branch migration activity on a HJ as well as replication fork regression, AtRECQ3 unwinds the lagging strand of replication forks and has a peculiar preference for the unwinding of nicked HJs [32,33]. The activity of both helicases on forked DNA substrates was analyzed in detail at single-molecule level and revealed further specificities of the two helicases. While both show a highly repetitive DNA helicase function, AtRECQ2 preferentially unwinds DNA but AtRECQ3 predominantly rewinds the DNA after short unwinding activity [34]. It is tempting to speculate that these different features also reflect specific in vivo functions of the two helicases. In yeast two-hybrid assays, an interaction of AtRECQ2 with the small exonuclease containing protein WRNexo was demonstrated [5]. As WRNexo depicts high homology to the exonuclease domain of human WRN helicase and similar biochemical features, it was hypothesized that RECQ2 and WRNexo form the functional homolog of HsWRN [35]. Based on its biochemical characteristics and domain composition, AtRECQ3 was proposed to be most likely the *Arabidopsis* homolog of HsRECQ5β. However, recent analyses of AtRECQ2 and AtRECQ3 did not reveal any in vivo function of the two helicases, as mutant lines were viable without growth defects, fully fertile and did not exhibit sensitivity against crosslinking agents [36]. This does not necessarily mean that the two helicases do not possess cellular features but possibly that they act as backups and their function only becomes apparent with the simultaneous mutation of multiple RecQ helicases. 

Exciting peculiar functions could be demonstrated for the RecQ1 homolog from rice. OsRECQ1 was initially characterized by expression analyses together with OsRECQ2, OsRECQ6 and OsRECQsim. Thereby, the expression of all four RecQ homologs was shown to be induced by MMS and bleomycin, furthermore OsRECQ1 expression was strongly induced by the crosslinker MMC [37]. Further analyses revealed an involvement of OsRECQ1 in RNA silencing induced by particle bombardment. Here, a role of OsRECQ1 in the transcription of inverted repeat DNA into dsRNA was postulated [38]. This connection of OsRECQ1 with RNA silencing was sustained by findings hinting to a function in the biogenesis of small interfering RNAs. The induction of a specific class of small RNAs and their precursors by treatment with genotoxins were shown to be dependent on OsRECQ1. Furthermore, increased cell death and defects in root growth could be detected in this mutant line [39]. This hints to a specific role of OsRECQ1 in DNA repair which might or might not be directly linked with small RNA induction. Whether such a function is conserved in other RecQ1 homologs in plants and what exact biological role RecQ1 has, remains to be elucidated in the future. 

RecQ6 homologs are not present in all plant clades and possess only low homology to the other RecQ subfamilies, thus they were proposed to be of ancient origin and then lost in *Brassicales* and *Malvales* during evolution [1]. Recently, the RecQ6 homolog from *P. patens* was characterized in detail. No function for PpRECQ6 in DNA repair could be detected, however an important role of the protein in HR was shown. Interestingly, PpRECQ6 seems to act as an enhancer of GT, as GT frequency was drastically reduced in ΔPp*RECQ6* lines [2]. Recent research in bread wheat identified a RecQ6 homolog (RECQ7) that is involved in promoting meiotic gene conversion, as the knock-out of RECQ7 leads to a reduction of gene conversion frequency in correlation with a decrease in CO frequency [40]. This hints to conserved features of the plant-specific RecQ6 homologs in promoting recombination. RECQsim homologs were proposed to originate from the RecQ6 subfamily, however they differ strikingly from other RecQ helicases by an insertion in the helicase domain [1]. A cross-kingdom complementation study demonstrated that expression of AtRECQsim is able to rescue the MMS sensitivity of *Saccharomyces cerevisiae sgs1* mutants [41]. This implies a conserved helicase function of AtRecQsim despite its special domain structure. However, what role RecQsim might fulfil in plants is still elusive. 

### 2.3. HRQ1

Apart from the classical RecQ homologs, plants and fungi harbor an additional RecQ-like helicase. The helicase HRQ1 (homologous to RecQ helicase 1) was identified during a search for HsRECQ4 homologs [42]. Similar to the other members of the RecQ helicase family, Hrq1 from *Saccharomyces cerevisiae* was demonstrated to act as 3’–5’ helicase, furthermore an involvement in CL repair in a common pathway with nucleotide excision repair (NER) was postulated [43,44,45]. The HRQ1 homolog from *Arabidopsis* was recently characterized. AtHRQ1 seems to fulfil a central role in CL repair, where the helicase is acting in a common pathway with AtRECQ4A (with RECQ4A as main RecQ helicase) in intrastrand CL repair. In ICL repair, a parallel involvement of both AtRECQ4A and AtHRQ1 is reminiscent to the situation in baker’s yeast with the involvement of Sgs1 and Hrq1 [36,46]. AtHRQ1 furthermore shares a common pathway with the Fanconi anemia-associated nuclease FAN1. Interestingly, an involvement of HRQ1 in post-replicative DNA repair (PRR) was postulated, which seems to be plant-specific [36].

## 3. Fe-S Cluster Helicases

The DNA helicase family of Fe-S cluster helicases was named after the conserved metal binding domain in the helicase core [47]. Mutations in the human Fe-S cluster helicases XPD, CHL1, FANCJ and RTEL1 are associated with severe hereditary diseases, highlighting the importance of this helicase family for the maintenance of genome stability [48]. Homologs of all four helicases have been identified in plants [49,50]. Here we will focus on the two closely related helicases RTEL1 and FANCJ as recent research unveiled interesting features in safeguarding genome stability in plants.

### 3.1. RTEL1

The Fe-S cluster helicase RTEL1 (regulator of telomere elongation helicase 1) is a main player in sustaining genome stability in multiple ways. This reflects in the appearance of Hoyeraal–Hreidarsson syndrome, a severe form of dyskeratosis congenital in humans upon mutation of *RTEL1* [51,52,53]. RTEL1 performs important roles as a antirecombinase, as it counteracts HR by disassembling D-loops [54]. For *C. elegans* RTEL1, an involvement in the repair of DNA damage like ICLs blocking replication fork progression was demonstrated. However, the most remarkable characteristic of mammalian RTEL1 is its function in telomere homeostasis, already leading to the identification of RTEL1 in mouse species exhibiting different telomere lengths [55,56]. Mechanistically, RTEL1 was shown to dismantle T-loops and G4 structures associated with telomeres [57]. The characterization of the *Arabidopsis* homolog of RTEL1 confirmed conserved functions of the helicase in plants [50,58]. Thereby, AtRTEL1 suppresses homologous recombination and is involved in the repair of CLs and replication-associated DNA damage in the root meristem. Interestingly, the concurrent loss of RTEL1 and RECQ4A triggers drastic developmental defects leading to degenerated roots, fasciated shoots, reduced size and sterility. This hints to essential roles of both helicases, likely acting on similar recombination intermediates in independent ways [50]. It seems probable that RECQ4A acts in the conserved RTR-complex, as double mutants of *RTEL1* with the complex partner *RMI2* depict similar, but less drastic, restrictions in root growth and fertility [25]. Moreover, *rtel1* mutants show a prolonged S-phase and activated cell cycle checkpoints [58]. The basic functions of RTEL1 in DNA repair seem to be conserved among plants, as knock-out lines of the *P. patens RTEL1* homolog depicted strong growth deficiencies and hypersensitivity against bleomycin and UV irradiation [59,60].

The most interesting detail however is that RTEL1 homologs in plants play multiple roles in stabilizing repetitive DNA sequences. The telomeric role of RTEL1 is conserved in plants, as the simultaneous absence of RTEL1 and telomerase TERT in *Arabidopsis* causes an accelerated shortening of telomere length, ultimately resulting in severe developmental defects after four generations [50]. The function of AtRTEL1 in telomere maintenance was further specified in an analysis that revealed a telomere-stabilizing role of RAD51-mediated HR. In the absence of telomerase, the telomeric RAD51 function was shown to rely on RTEL1, indicating a role for the helicase in processing recombination intermediates arising through RAD51 recombinase activity [61]. It was therefore postulated that RTEL1 and RAD51 act in an alternative lengthening of telomeres (ALT) mechanism to stabilize telomeres in the absence of TERT [62]. However, telomere length in At*rtel1* mutant lines is not affected when TERT is present [25,50]. Astonishingly, an important role for AtRTEL1 in the maintenance of another class of repetitive genomic sequences, the rDNA arrays, could be unveiled, that has so far not been described in any animal system. At*rtel1* mutant lines show a reduction of 45S rDNA repeat number to only half of the original number. The additional mutation of the RTR-complex partner *RMI2* lead to an even further decrease resulting in a third of the copies. At the same time, HR between chromosomal repeats is enhanced by almost two orders of magnitude [25]. The function of RTEL1 on repetitive sequences applies also to *P. patens* but with slight differences: telomere length was drastically affected in Pp*rtel1* mutant lines in the presence of TERT, possibly due to the high activity of HR in the moss which is further elevated in the absence of RTEL1 [60]. However, PpRTEL1 contributes to 45S rDNA stability in similar ways as in *Arabidopsis*, as a corresponding reduction in copy number could be determined in the respective mutant. This indicates conserved functions of the helicase in stabilizing repetitive genomic loci in plants, and raises the question for a similar role in other eukaryotes. Although no effect of RTEL1 on rDNA stability in animals has been reported as yet, this might be of high relevance, especially as it may also contribute to the understanding of genetic diseases.

### 3.2. FANCJ

**Figure 2:**
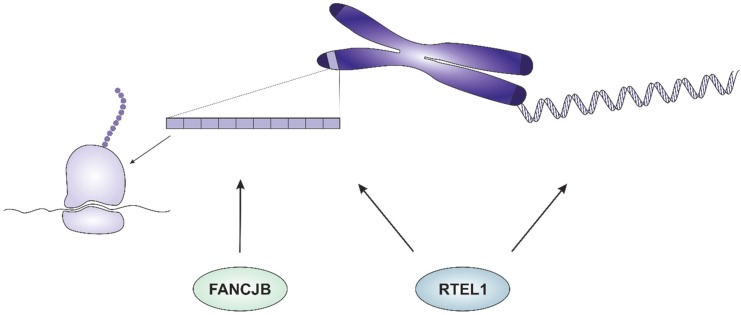
Fe-S cluster helicases involved in the stabilization of repetitive sequences in plants. The RTEL1 helicase is involved in the stabilization of both telomeric DNA (right) and rDNA repeats (left). FANCJB fulfils a parallel function to RTEL1 in the maintenance of rDNA repeat number.

Human FANCJ helicase is a member of the Fanconi anemia (FA) pathway, responsible for ICL repair. Mutation in the FA genes leads to the hereditary disease FA, characterized by congenital defects, bone marrow failure and a predisposition for cancer [63]. The first description of FANCJ identified it as a direct interaction partner of BRCA1 (therefore FANCJ was initially called BACH1 or BRIP1), and a common involvement in enhancing HR has been demonstrated [64,65]. Recently, an initial characterization of FANCJ homologs from *Arabidopsis thaliana* has been performed [13]. *Arabidopsis* harbors a duplicated gene pair of *FANCJ* homologs, called At*FANCJA* and At*FANCJB*. Interestingly, the At*FANCJ* genes are only separated by two genes on chromosome 1 and although both genes show high similarity and similar homology to human *FANCJ*, diverse functions could be determined. Consequently, only AtFANCJB seems to be the functional homolog to human FANCJ, as a role for AtFANCJB in ICL repair could be determined, which was not the case for AtFANCJA. In contrast to the closely related AtRTEL1, AtFANCJB does not fulfil any role in the suppression of HR, as recombination frequencies in At*fancjb* mutant lines correspond to wild type plants. However, the genetic interaction of both Fe-S cluster helicases turned out to be of special relevance. At*fancjb rtel1* double mutants depict an astonishing viable phenotype, in contrast to the synthetic lethality from animal systems [54]. Nevertheless, analyses in root meristems demonstrated independent roles for both helicases in replication-associated DNA repair. Most strikingly, double mutants revealed a reduction in 45S rDNA copy number to only one third of wild type plants. This implies independent functions for both helicases in stabilizing rDNA repeats, expanding the plant Fe-S cluster helicases involved in stabilizing repetitive sequences to FANCJB (Figure 2). It was postulated that the discovered instability arises due to a possible function of RTEL1 and FANCJB in unwinding G-quadruplexes (G4s) [13]. G4s consist of guanine tetrads, interacting with each other via Hoogsteen base pairing. Multiple stacked tetrads connected by DNA loops then build a G4 [66]. G4s can be formed in G-rich sequences like ribosomal DNA, and the correct course of all DNA-dependent processes rely on the efficient unwinding of such G4s, due to their stability. Therefore, the simultaneous loss of AtFANCJB and AtRTEL1 might lead to an accumulation of persisting G4s in the rDNA locus, hindering correct replication and ultimately leading to a loss of rDNA copies. This is further supported by analyses from *P. patens*, where a highly G4-prone sequence was detected in the rDNA locus [60]. Detailed knowledge regarding G4 DNA and its regulation in plants is still scarce, so this will be an exciting research field in the future, especially since many helicases were shown to be involved in G4 metabolism in other organisms [67].

## 4. Further Helicases Involved in Genome Maintenance

Multiple helicases from different families fulfil crucial roles in stabilizing genomes. As a result, they are involved in a multitude of pathways for DNA repair, recombination or replication each helping genome maintenance in independent ways. Unfortunately, in comparison to other eukaryotes, only a limited number of studies have been performed in plants. In the following section, we will focus on some prominent helicases known from other eukaryotes that have also been characterized in plants regarding their role in safekeeping the genome. 

### 4.1. SRS2

The SRS2 helicase is not present in mammals, but seems to be conserved across most other eukaryotes, including plants [68]. *Saccharomyces cerevisiae* Srs2 has been characterized in detail and was shown to fulfil important roles in DNA replication, recombination and repair [69]. Thereby, the most prominent function of Srs2 is as an antirecombinase in disrupting Rad51-ssDNA nucleoprotein filaments. For the SRS2 homolog from *Arabidopsis*, detailed in vitro analyses have been performed [70], whereby a conserved 3’–5’ helicase activity was demonstrated. Furthermore, related functions to yeast Srs2 were uncovered, as AtSRS2 processes branched DNA structures in a similar way. In doing so, the helicase unwinds partial HJs, resembling structures arising during synthesis-dependent strand-annealing (SDSA), indicating a possible in vivo function of the helicase in HR by promoting NCO outcomes. Astonishingly, AtSRS2 seems to possess additional features not described for the yeast homolog. The helicase was demonstrated to mediate strand annealing, suggesting a role in SDSA in the re-annealing of the displaced strand following elongation [70]. However, no defined organismic role for AtSRS2 could be identified yet. After all, SRS2 from *P. patens* is the only plant homolog characterized in vivo so far in any case. There, expression of Pp*SRS2* was shown to be highly induced by γ-irradiation, but mutants did not depict hypersensitivity against bleomycin. Furthermore, no influence of PpSRS2 on GT efficiency could be detected [59,71]. Thus, the function of SRS2 in plants remains elusive and whether the helicase contributes to genome maintenance in plants is still an open question. 

### 4.2. FANCM

Apart from the Fe-S cluster helicase FANCJ, another helicase associated with FA, FANCM, has been described to contribute significantly to genome stability in plants. Human FANCM helicase is the central player for ICL recognition in the FA pathway. FANCM initiates the pathway by recruiting the FA core complex, which then activates the FANCD2/I heterodimer. This leads to the recruitment of additional downstream factors for the repair of the ICL [63]. Amongst the core FA factors, FANCM is also the only one possessing a functional homolog in yeast, named Mph1. The plant FANCM homolog was shown to support genome stability on multiple levels. AtFANCM possesses antagonistic functions in somatic HR. Thereby, naturally occurring HR is upregulated in At*fancm* mutant lines, while the HR rate is reduced after bleomycin induction, indicating a HR promoting function of FANCM after DSBs, while it suppresses spontaneously occurring HR. Interestingly, AtFANCM and the RecQ helicase AtRECQ4A were shown to act in independent pathways in the suppression of spontaneous HR [72]. Further studies specified the role of AtFANCM in HR, where it was demonstrated to be involved in promoting SDSA, but also SSA to a minor extent [73]. Astonishingly, the role of AtFANCM in ICL repair is minor in plants in contrast to its human homolog, since At*fancm* single mutants do not exhibit hypersensitivity against crosslinking agents [72]. A conserved function for AtFANCM in ICL repair could only be revealed in double mutant analyses when other CL repair factors were also knocked out. Therefore, a parallel function of AtFANCM and AtRECQ4A in ICL repair could be confirmed, matching the independent roles of both helicases in HR. Furthermore, FANCM was demonstrated to possess an essential role in the repair of replicative DNA damage in the absence of the MUS81 endonuclease, reflected in the synthetic lethal phenotype of double mutant lines [11]. However, the most striking characteristic of FANCM in plants is its role in meiotic recombination. The initial characterization of AtFANCM hinted to a function of the helicase in suppressing meiotic CO formation. Thereby, the loss of FANCM leads to meiotic aberrations, however this is combined with a 3-fold increase in CO frequency [72,74]. AtFANCM was shown to specifically suppress interference-insensitive COs, arising through a MUS81-dependent pathway. Further analysis identified the two FANCM-associated proteins AtMHF1/2 to participate in this function, while no other FA protein shared similar characteristics [75]. As plants lack the classical FA pathway, it is not surprising that FANCM and its cofactors play a discrete role in meiotic CO formation. However, this seems to be a function conserved at least in *Brassicaceae*, as a similar CO-suppressing capacity of FANCM was found in *Brassica rapa* and *Brassica napus* [76]. The meiotic role of AtFANCM was further specified whereby the CO elevation following *FANCM* mutation was shown to be restricted to inbred lines, whilst it was not apparent in hybrids [77,78]. The number of meiotic COs can be even further elevated in the absence of both FANCM and RECQ4A/B. Thus, it was postulated that both helicases independently limit CO formation. Consequently, around half of the DSBs induced during meiosis result in a CO. It was suggested that the helicases might act on different kinds of substrates within the SDSA of meiosis [19]. The simultaneous loss of FANCM and RECQ4A/B eliminates SDSA during meiosis, leaving all meiotic recombination intermediates to be resolved by nucleases. An unbiased action of these resolvases would result in a CO product in roughly half of all cases. This massive unleashing of CO recombination has high potential for breaking linkages more efficiently in plant breeding (see Section 5.2.).

### 4.3. MCM Helicases

The heterohexameric complex of minichromosome maintenance proteins 2-7 (MCM2–7) acts both in the licensing and initiation of replication and as replicative DNA helicase. Therefore, they are essential for genome stability. Both the initiation of replication has to be strictly regulated and the efficient unwinding of the DNA must be ensured [79]. The importance of MCM proteins for correct DNA replication in plants is reflected by the embryo lethality of At*mcm2* mutants. Furthermore, the overexpression of At*MCM2* has an inhibitory effect on endoreduplication, hinting to the important role of MCM proteins for safeguarding replication regulation [80]. Further analyses hinted to subtle differences in the function of the distinct MCM2–7 subunits during seed development. Although the expression of the individual subunits seems to be coordinated, and every subunit is necessary for correct seed development, MCM7 was suggested to possess an exceptional role in the egg cell. Depletion of the MCM2–7 complex induced an upregulation of DNA repair genes, indicating an accumulation of DNA damage due to its improper functioning [81]. A detailed biochemical characterization of AtMCM3 could confirm ATPase and DNA unwinding activity. The helicase hereby acts in the 3’–5’ direction possibly as a multimer [82]. Interestingly, also for MCM6 from pea, an ATP dependent 3’–5’ helicase activity could be demonstrated, highlighting the protein forms a homohexamer [83]. The activity of single MCM subunits as functional helicases might be a unique feature for plants, as this was not reported for homologs from other eukaryotes. Astonishingly, the overexpression of Ps*MCM6* in tobacco confers salinity resistance, hinting to an important role of the MCM proteins in stress tolerance [84]. This knowledge could potentially be exploited in the generation of stress tolerant crop plants, adding a whole new aspect to helicase research [85]. Although the MCM2–7 proteins are highly conserved throughout eukaryotes, the presence of another MCM protein, MCM8 is restricted to vertebrates and plants. MCM8 proteins seem to fulfil a special role in meiotic recombination. For AtMCM8, a function in the repair of meiotic DSBs has been demonstrated, as mutant lines exhibit chromosomal fragmentation during meiosis, without affecting CO number. It was suggested that AtMCM8 thereby acts in parallel to DMC1, in a backup pathway with RAD51, resulting in NCO outcomes [86]. This further highlights the extremely diverse functions of this seemingly uniform protein family. 

### 4.4. POLQ/Θ (TEBICHI)

DNA polymerase θ (POLQ) is a unique kind of protein, as it combines a C-terminal DNA polymerase and a N-terminal DNA helicase domain in a single gene product. This peculiar assembly also reflects in the involvement of POLQ in multiple exceptional pathways. POLQ is highly conserved throughout multicellular eukaryotes and performs essential functions in DSB repair via microhomology-mediated end-joining (MMEJ) [87]. POLQ generates a distinct repair pattern consisting of indels at the repaired break site and microhomologies flanking it [88,89]. Interestingly, the POLQ helicase domain exhibits similarities to the RecQ helicase family and for human POLQ, both DNA polymerase and 3’–5’ DNA unwinding activity could be demonstrated [87,90,91,92]. POLQ homologs are also present in plants and the *Arabidopsis* homolog was originally termed TEBICHI (TEB). This is linked to the initial characterization, as *tebichi* is a Japanese name for the cloven hoof of a pig and At*teb* mutants were shown to exhibit reminiscent morphological defects [93]. Nevertheless, for the sake of clarity, we will use the following term AtPOLQ to refer to this enzyme. For the helicase and polymerase domains of AtPOLQ, a high degree of conservation to other eukaryotic homologs could be determined. Astonishingly, only *Arabidopsis* mutants harboring disruptions in the N-terminal helicase domain exhibited growth defects, while this was not the case for defects in the C-terminal polymerase domain [93]. This further underlines a possible important contribution of the helicase function of AtPOLQ to its different tasks in the cell. Furthermore, AtPOLQ was shown to be involved in DNA repair, as mutant lines exhibit hypersensitivity against the crosslinkers MMC and cisplatin, as well as the methylating agent MMS [93,94]. Further analysis identified a parallel involvement of AtPOLQ and the PRR translocase AtRAD5A in DNA repair [94]. Subsequently, POLQ was postulated to act in parallel to HR in replication dependent DNA repair, matching the role of POLQ in MMEJ or possibly being linked to a function of POLQ as a translesion polymerase. This parallel involvement of POLQ with HR is further highlighted with the enhanced growth defects in *polq* mutants additionally lacking the HR-related factors XRCC2 or RAD51D [95]. Moreover, a genetic interaction of AtPOLQ with the repair kinase AtATR, responsible for the DNA damage response to replication stress, could be determined [95]. For the POLQ homolog from *P. patens*, an increased expression after DSB-induction was shown, confirming the conserved function of plant POLQ homologs in DSB repair [59]. However, Pp*polq* mutants do not exhibit hypersensitivity against MMS or cisplatin, possibly justified by the different balance of DSB repair by HR or end-joining mechanisms in the moss [96]. Nevertheless, a critical role of PpPOLQ in the repair of CRISPR/Cas9 induced DSBs could be determined, as the CRISPR-induced mutation rate in Pp*polq* mutants is reduced and remaining mutations do not rely on MMEJ. Additionally, GT was strongly enhanced in the absence of POLQ, further highlighting the important role of POLQ and MMEJ as antagonists to HR [96]. The most astonishing function of POLQ in plants is probably its essential role in T-DNA integration. Agrobacterium mediated plant transformation via the transfer and random integration of a T-DNA into plant genomes is an indispensable tool for basic research and biotechnological applications [97]. The exact mechanism of T-DNA integration was unclear until an involvement of POLQ was discovered in 2016 [98]. It could be demonstrated that At*polq* mutants are susceptible to Agrobacterium infection, but completely deficient in T-DNA integration. Moreover, junctions between the 3’ end of the T-DNA and the plant genome revealed patterns matching with the action of POLQ, such as the presence of microhomologies as well as filler sequences. Thus, T-DNA integration would closely resemble MMEJ with the T-DNA as partner molecule. This startling finding is of high relevance for biotechnological applications, possibly enabling new strategies for the generation of transgenic crops.

## 5. Helicases are Relevant for Multiple Aspects of Plant Breeding

**Figure 3:**
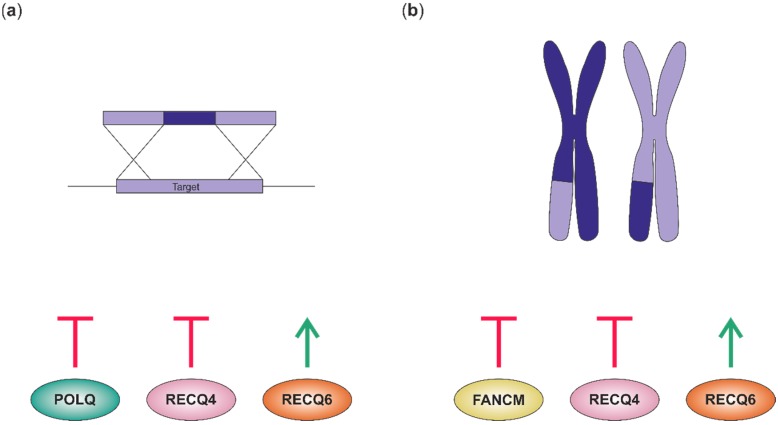
Plant helicases and their impact on gene targeting and crossover formation. (**a**) Gene targeting efficiency in *P. patens* is repressed by POLQ and RECQ4, while RECQ6 has a stimulating effect. (**b**) Crossover (CO) formation is inhibited by FANCM and RECQ4, demonstrated for a wide number of plant species. In bread wheat, RECQ6 was additionally identified as CO promoting factor.

The knowledge gained from studies concerning DNA helicases is not only of high value for basic research, but might also contribute to specific applications. As climate change and a growing world population challenge food security, breeders have to face these obstacles with new tools. Possible new ways to improve plant traits can be derived from recent findings arising through helicase research. In the following sections we want to highlight how helicases influence GT and CO frequency in plants (Figure 3) and how this might facilitate certain aspects of breeding.

### 5.1. Gene Targeting

Gene targeting (GT) is a technique that holds outstandingly high potential for plant breeding. With GT, it is possible to modify the genome precisely through HR. Thereby, genetic information can be inserted or substituted accurately at a specific location using a homologous template [99]. However, as error-prone non-homologous end-joining is the dominant DNA repair pathway in higher plants and HR activity is very low, this impedes the application of GT due to low frequencies. Therefore, manipulating DNA repair pathways to shift the equilibrium towards HR promises to improve GT efficiency. As helicases are of central importance for DNA repair reactions, they are prime candidates for manipulation aiming to upregulate GT. Interesting results were obtained with the moss *Physcomitrella patens*. Two RecQ helicases PpRECQ4 and PpRECQ6 were demonstrated to possess antagonistic functions in modulating GT [2]. Disruption of the RecQ helicase PpRECQ4 enhanced GT frequency from 66% to an astonishing 94%, implying a strong GT suppressing function of PpRECQ4. In contrast to this, PpRECQ6 was postulated to act as a potent enhancer of GT, since GT frequency in *ΔPpRECQ6* lines was drastically reduced to 15%. This raises the question of possible overexpression analyses of PpRECQ6 for an enhancement of GT. However, as basic GT efficiency in *P. patens* in comparison to *Arabidopsis* is enhanced by at least one order of magnitude, these findings are still difficult to apply to higher plants. It is noticeable that *Arabidopsis* does not harbor a RecQ6 homolog. Thus, it might be interesting to test whether the expression of a heterologous RecQ6 protein would be able to enhance GT efficiencies in *Arabidopsis*. 

It was furthermore shown that not every approach of enhancing chromosomal HR in *Arabidopsis* does necessarily correlate with an enhancement of GT frequency. *Arabidopsis* plants with a simultaneous mutation in the Fe-S cluster helicase *RTEL1* and the RTR complex partner *RMI2* depict a strong hyperrecombination phenotype, with a spectacular 80-fold elevation in chromosomal HR [25]. Another interesting double mutant line where *RTEL1* in combination with the FA helicase *FANCM* is affected, exhibits a still impressive 20-fold chromosomal HR increase [50]. However, CRISPR/Cas mediated GT in both double mutant lines using the in planta GT approach did not result in any increase in GT rate [100,101]. Thus, there is no simple way to transfer knowledge from chromosomal HR pathways to GT applications. Another promising approach to enhance GT utilizes the MMEJ factor POLQ. The deletion of *POLQ* in *P. patens* increased GT frequency to an amazing 98% [96]. However, as *Arabidopsis* plants deficient in the POLQ homolog TEBICHI are resistant towards stable agrobacteria-mediated transformation, it is more difficult to apply this approach in higher plants [98]. As a result, although multiple interesting concepts on the utilization of helicases in enhancing GT frequencies in plants do exist, no general solution to the problem has been developed as yet.

### 5.2. Manipulation of CO Frequency

The basis of plant breeding approaches relying on the natural variability inherent in different individuals, is the efficient exchange of their genetic information during meiosis. Here, recombination leads to COs, the reciprocal exchange between parental chromosomes. However, CO formation in plants is tightly regulated to both a limited number and preferred regions in the genome. Therefore, it is of high interest for plant breeders to influence CO frequency and distribution to enable the breaking of linkages and the generation of new beneficial genetic combinations [102,103,104]. The helicases RecQ4 and FANCM have been shown to be involved in CO regulation in plants. Initial studies from *Arabidopsis* demonstrated strong anti-CO functions for both *FANCM* and the *RECQ4A/B* gene pair. In *fancm* mutant lines, CO frequency was elevated 3-fold, while the *recq4a/b* double mutant depicts an astonishing 6-fold CO elevation [19,74]. The combination of both in a triple mutant further increased this to a 9-fold boost [19]. The transfer of this knowledge to crops was performed in an initial analysis, testing the inactivation of *FANCM* homologs in Brassica crops. Thereby, in *fancm* EMS mutants from *Brassica rapa* and *Brassica napus*, a 3-fold and 1.3-fold CO number increase could be confirmed, respectively. However, the demonstrated increase in *Brassica rapa* referred to a *msh4* mutant line and not wild type. It was furthermore postulated that the lower increase in *Brassica napus* depended on the residual activity of one of the *FANCM* copies. However, the measured enhancement in *Brassica napus* was present in both allotetraploid and allohaploid plants, indicating a rise in CO frequency not only between homologous but also homeologous chromosomes [76]. This is of high relevance as the CO elevation by *fancm* mutation was shown to be absent in *Arabidopsis* hybrids [77,78]. An alternative option for increasing CO frequency in a hybrid context might be the combination of a mutation in the RecQ helicases *RECQ4A/B* and the AAA-ATPase *FIGL1*, which resulted in a nearly 8-fold CO boost in *Arabidopsis*, while the *recq4a/b* double mutant alone already depicted an almost 4-fold increase [105]. The further application of this helicase-based approach in crop plants was demonstrated in rice, pea and tomato [21]. Thereby, the mutation of *FANCM* leads to a 2-fold CO increase in both hybrid rice and hybrid pea, standing in contrast to the results from *Arabidopsis*. The extraordinary role of RecQ4 homologs in CO-suppression seems to be a conserved feature in plants and mutating them could be a universal tool in enhancing meiotic recombination in crops. The mutation of *RecQ4* homologs resulted in a rise of CO-frequency in rice (3.2 ×), pea (4.7 ×) and tomato (2.7 ×) hybrids, promising exciting utilization of this approach for breeding, especially since the obtained lines were fully fertile, with only a minor reduction of fertility in pea [21]. Indeed, the further applicability of this approach was recently proven in an interspecific *Solanum lycopersicum* and *S. pimpinellifolium* hybrid, harboring biallelic CRISPR/Cas9-induced *RECQ4* mutations, where a 1.8-fold rise in COs could be demonstrated. This lays the path for transferring beneficial traits from wild relatives to crops in an accelerated manner. The search for CO regulators in plants is not exhausted yet, as a study from bread wheat identified a RecQ6 homolog (RECQ7) as an important QTL affecting gene conversion frequency. Wheat EMS *recq7* mutants exhibited a reduced gene conversion frequency, combined with a decrease in CO frequency, indicating important roles for this helicase in enhancing recombination [40]. As no classical RecQ6 homolog is present in *Arabidopsis*, this further highlights the limitations of model plants and the need for expanding research to individual crop species, now achievable through gene editing techniques. Thus, manipulating DNA helicases might be an important contribution for plant breeding. However, a note of caution is necessary as all these helicases are involved in ensuring genome stability in somatic cells: Changes in the machinery might also result in further unwanted genome instabilities in somatic cells, which might endanger the applicability of the technology within the field. Thus, further research is necessary to address this point in detail. 

## 6. Conclusion and Perspectives

Recent research on DNA helicases as the guardian angels of genome integrity in plants has revealed a number of interesting new results. Besides conserved basic functions with other eukaryotes, a variety of interesting new features have been described for plants. The necessity of these important proteins for all DNA-based cellular processes is reflected in the human hereditary diseases associated with the loss of specific helicases. Current research in plants could highlight new roles for these helicases in the maintenance of repetitive DNA sequences and CO formation during meiosis, which might also be relevant for human disease. Furthermore, the manipulation of helicase genes opens new avenues for plant breeders. Thus, improvements of CO frequency and CRISPR/Cas mediated gene targeting should be achievable. Therefore, further research on helicases, not only in model plants like *Arabidopsis* but also crops, harbors great potential for further improving breeding techniques. Paired with the recent progress in gene editing, this holds the promise for exciting new applications possibly contributing to the future assurance of food security. 

## Figures and Tables

**Table 1:** RecQ helicase family in plants. The table gives an overview about the six RecQ helicase subfamilies and their respective representatives in humans, *Arabidopsis thaliana* and *Physcomitrella patens* [2].

RecQ Subfamily	Human	*Arabidopsis thaliana*	*Physcomitrella patens*
RecQ1	-	RECQ1	-
			
RecQ2	RECQ1	RECQ2	RECQ2
			
RecQ3	RECQ5	RECQ3	-
			
RecQ4	BLM	RECQ4ARECQ4B	RECQ4
			
RecQ5	RECQ4	RECQ5	RECQ5
		(HRQ1)	
RecQ6/RecQsim	WRN	RECQsim	RECQ6 RECQsim1 RECQsim2
